# Long-Term Oxygen Therapy 24 vs 15 h/day and Mortality in Chronic Obstructive Pulmonary Disease

**DOI:** 10.1371/journal.pone.0163293

**Published:** 2016-09-20

**Authors:** Zainab Ahmadi, Josefin Sundh, Anna Bornefalk-Hermansson, Magnus Ekström

**Affiliations:** 1 Department of Clinical Sciences, Division of Respiratory Medicine & Allergology, Lund University, Lund, Sweden; 2 Department of Respiratory Medicine, School of Medical Sciences, Örebro University, Örebro, Sweden; 3 Department of Statistics, Uppsala University, Uppsala, Sweden; National and Kapodistrian University of Athens, GREECE

## Abstract

Long-term oxygen therapy (LTOT) ≥ 15 h/day improves survival in hypoxemic chronic obstructive pulmonary disease (COPD). LTOT 24 h/day is often recommended but may pose an unnecessary burden with no clear survival benefit compared with LTOT 15 h/day. The aim was to test the hypothesis that LTOT 24 h/day decreases all-cause, respiratory, and cardiovascular mortality compared to LTOT 15 h/day in hypoxemic COPD. This was a prospective, observational, population-based study of COPD patients starting LTOT between October 1, 2005 and June 30, 2009 in Sweden. Overall and cause-specific mortality was analyzed using Cox and Fine-Gray regression, controlling for age, sex, prescribed oxygen dose, PaO_2_ (air), PaCO_2_ (air), Forced Expiratory Volume in one second (FEV_1_), WHO performance status, body mass index, comorbidity, and oral glucocorticoids. A total of 2,249 included patients were included with a median follow-up of 1.1 years (interquartile range, 0.6–2.1). 1,129 (50%) patients died and no patient was lost to follow-up. Higher LTOT duration analyzed as a continuous variable was not associated with any change in mortality rate (hazard ratio [HR] 1.00; (95% confidence interval [CI], 0.98 to 1.02) per 1 h/day increase above 15 h/day. LTOT exactly 24 h/day was prescribed in 539 (24%) patients and LTOT 15–16 h/day in 1,231 (55%) patients. Mortality was similar between the groups for all-cause, respiratory and cardiovascular mortality. In hypoxemic COPD, LTOT 24 h/day was not associated with a survival benefit compared with treatment 15–16 h/day. A design for a registry-based randomized trial (R-RCT) is proposed.

## Introduction

Long-term oxygen therapy (LTOT) improves survival time in patients with hypoxemic chronic obstructive pulmonary disease (COPD) when given for 15 h/day or more.[1, 2] LTOT is common and is associated with considerable logistics and costs.[3, 4] More than one million people use LTOT in the USA alone,[3] and the incidence is projected to increase in coming decades.[5] Morbidity and mortality are high despite LTOT, with a median survival of less than 2 years after start of oxygen therapy.[6] Strategies to improve prognosis in hypoxemic COPD are needed.

Although current guidelines state that LTOT should be provided continuously for 24 h/day,[7] data are limited on whether LTOT 24 h/day provides additional survival benefit compared with treatment 15 h/day.[1, 2, 8, 9] The recommendation of LTOT 24 h/day is based on an unadjusted comparison of the treatment arms of two randomized trials from the 1970s,[8] which showed that the mortality rate was lower for the 18 h/day group in one study than for the 15 h/day group in another study.[1, 2, 8] However, the effects of each LTOT arm compared to controls (12 h/day and no treatment respectively) were similar.[1, 2] The comparison was non-randomized and did not adjust for potential confounders.[8] Furthermore, most patients starting LTOT on therapeutic indication nowadays are women, elderly with multimorbidity and median survival less than two years, as compared to the original trials which might decrease the validity of the original RCT findings for current practice.[5, 9–12] Evidence from randomized trials whether continuous LTOT duration 24 h/day provides an additional survival benefit above 15 h/day is lacking.[9]

LTOT 24 h/day might pose an unnecessary burden for many patients compared with treatment 15 h/day where they can be unconnected to the machine for 9 hours each day.[13, 14] Supplemental oxygen therapy has been consistently associated with feelings of dependence, anxiety, guilt and shame among patients and caregivers, which could contribute to increased social isolation and restrictions of activity and daily life.[13, 15–17]

The aim was to test the hypothesis that LTOT 24 h/day compared with LTOT 15–16 h/day and adjusting for potential confounders decreases all-cause, respiratory, and cardiovascular mortality in hypoxemic COPD.

## Materials and Methods

### Design and population

This was a prospective national observational study of patients starting LTOT on therapeutic indication for physician-diagnosed COPD in the National Register for Respiratory Failure (Swedevox) between October 1, 2005 and June 30, 2009. Swedevox has a population-based coverage of approximately 85% of patients starting LTOT in Sweden since 1987.[18] Details of the register were described in a recently published study using the same database.[11] All clinics prescribing LTOT in Sweden have agreed to observe the guidelines from the Swedish Respiratory Society that LTOT should be given for 15 h/day or more.[19–21] Nationwide, 48 clinics are prescribing LTOT.[6] LTOT is mainly prescribed by a pulmonologist or an internist with special training in COPD and respiratory failure.[6] The indication criteria for LTOT are: COPD and resting hypoxemia for at least three weeks despite optimal management of the underlying disease(s). The required level of resting hypoxemia breathing ambient air is defined as an arterial blood gas tension of oxygen (PaO_2_) < 7.4 kPa; or PaO_2_ 7.4 − 8.0 kPa together with signs of right-sided heart failure/pulmonary hypertension and/or secondary polycythemia (erythrocyte volume fraction, EVF > 0.54). LTOT is titrated with the goal to obtain a PaO_2_ > 8 kPa or oxygen saturation > 90% on oxygen.[1, 2, 22, 23] LTOT daily duration prescribed may differ due to local traditions and based on patient-physician agreements in order to increase patient compliance when taking in regards patients’ special needs and preferences.

For patients who started LTOT more than once (N = 62) during the period, only the most recent treatment episode was included in the analysis. Exclusion criterion was a diagnosis of lung cancer before starting LTOT.[11]

### Data

Swedevox contains data on resting arterial blood gas tension of oxygen (PaO_2_) and carbon dioxide (PaCO_2_) breathing air and during oxygen therapy, forced expiratory volume in one second (FEV_1_), measured body mass index (BMI), smoking history and WHO performance status registered at the start of LTOT.[6] The prescribed daily dose (l/min) and duration (h/day) of LTOT are registered by a responsible specialized oxygen nurse at the time of starting LTOT.

Data on comorbidity during the four-year period before baseline were obtained from the National Patient Register for in- and outpatient care, which covers more than 99% of all admissions in the study period and about 80% of all hospital based outpatient care since 2001 in Sweden.[24] Diagnoses were coded according to the ninth (before 1997) [25] and tenth revisions of the International Classification of Disease (ICD),[26] as previously described.[27] Data on all dispensed drug prescriptions in outpatient care after July 1, 2005 were obtained from the Swedish Prescribed Drug Register.[28] Vital status and cause of death were obtained from the Swedish Causes of Death Register.

Patients were prospectively followed until the first of LTOT withdrawal, death, or study end December 31, 2009. The primary endpoint was death from all causes. Secondary endpoints were mortality from respiratory disease (ICD-10, J00–J99) or cardiovascular disease (ICD-10, I00–I99) as underlying cause of death.

### Ethical considerations

All patients participating in the study were informed according to directives from the authorities. Participants provided their verbal consent when registered in Swedevox and the consent procedure and the study was approved by the Lund University research ethics committee (DNr 157/2007 and 350/2008), the Swedish National Board of Health and Welfare, and the Swedish Data Inspection Board.

### Statistical analyses

Baseline patient characteristics were summarized using mean with standard deviation (SD) and median with range or interquartile range (IQR) for continuous variables with normal and skewed distribution, respectively. Categorical variables were expressed as frequencies and percentages.

All-cause mortality was analyzed using Cox regression and expressed as hazard ratios (HR). Cause-specific mortality was analyzed using Fine-Gray regression accounting for the competing risk of death from other causes,[29] and expressed as subdistribution hazard ratios (SHR).[29] All associations were calculated with 95% confidence intervals (CIs). The observation time was from the start date of LTOT until the date of death from all causes, with censoring at withdrawal of LTOT or 31 December 2009.

Covariates to be included in the final model were selected using subject matter knowledge and prior mortality analyses.[11, 27, 30] Missing elements were imputed for PaO_2_ (air) (N = 289), PaCO_2_ (air) (N = 301), FEV_1_ (N = 849), body mass index (BMI) (N = 701) and WHO performance status (N = 199), as previously described.[11] The model estimates were robust to the imputations.

The final models were adjusted for baseline age, sex, prescribed oxygen dose, PaO_2_ (air), PaCO_2_ (air), FEV_1_, WHO performance status, BMI categories, maintenance treatment with oral glucocorticoids and comorbid diseases in terms of anxiety, renal failure and number of cardiovascular diagnoses (cerebrovascular disease, heart failure, hypertension, ischemic heart disease, peripheral artery disease, pulmonary embolism, and other circulatory disease). The primary analysis was among all patients (N = 2,249) with the prescribed daily LTOT duration analyzed as a continuous variable (h/day). In a secondary analysis, a comparison of LTOT prescribed for 24 h/day versus 15–16 h/day was carried out including only patients with either LTOT prescription (N = 1,770). Statistical significance was defined as two-sided p-value < 0.05. The differences among the groups were tested with t tests for continuous and chi-square tests for categorical variables. Statistical analyses were conducted using the software packages Stata, version 13 (StataCorp LP; College Station, TX), and SAS, version 9.3 (SAS Institute, Inc., Cary, NC).

## Results

### Patient characteristics

A total of 2,249 patients (59% women) started LTOT for COPD during the study period and were included in the main analysis. During a median follow-up of 1.1 years (IQR, 0.6 to 2.1 years), 138 (6%) patients withdrew from LTOT mainly because of improved oxygenation, and 1,129 (50%) patients died. The median survival time was 1.9 years (IQR, 0.7 to 4.0 years). Main causes of death included respiratory disease (68%), cardiovascular disease (20%) and cancer (6%).

In the cohort, 539 (24%) patients were prescribed LTOT 24 h/day, 1,231 (55%) were prescribed 15 h/day and 470 (21%) had other daily durations prescribed. In the LTOT 24 h/day group, 288 (53%) patients died and 629 (52%) patients died in the LTOT 15–16 h/day group. Compared with the LTOT 15–16 h/day group, patients with LTOT 24 h/day had worse functional status (Table 1). PaO_2_ on oxygen above 8 kPa was achieved in the majority of patients, with similar rates for LTOT 24 h/day (77%) and 15–16 h/day (80%).

**Table 1:** Baseline characteristics in oxygen-dependent chronic obstructive pulmonary disease patients.

Characteristic	All on LTOTN = 2,249	LTOT 24 h/dayN = 539 (24%)	LTOT 15–16 h/dayN = 1,231 (55%)	P-value
Age, years	74.7 ± 8.2	75.0 ± 8.1	74.7 ± 8.2	0.57
Women, n (%)	1,328 (59)	283 (53)	767 (62)	< 0.001
PaO_2_ air, kPa	6.5 ± 0.9	6.3 ± 0.9	6.6 ± 0.8	< 0.001
PaO_2_ oxygen, kPa	8.7 ± 1.1	8.6 ± 1.1	8.7 ± 1.1	0.006
PaCO_2_ air, kPa	6.3 ± 1.2	6.2 ± 1.3	6.2 ± 1.2	0.90
PaCO_2_ oxygen, kPa	6.5 ± 1.3	6.6 ± 1.3	6.5 ± 1.3	0.12
FEV_1_, L	0.84 ± 0.48	0.89 ± 0.54	0.83 ± 0.45	0.10
FEV_1_, % of predicted	33.6 ± 17.3	34.8 ± 19.5	33.8 ± 17.0	0.45
Prescribed Oxygen dose, L/min	1.6 ± 1.3	2.0 ± 1.3	1.5 ± 1.0	< 0.001
Ever smoking, n (%)	2,106 (94)	478 (89)	1110 (90)	0.35
Body mass index, kg/m^2^	24.0 (6.3)	24.0 (6.4)	23.9 (6.0)	0.80
WHO performance status, n (%)				
0	132 (6)	26 (5)	76 (6)	0.26
1	881 (39)	173 (32)	493 (40)	0.001
2	714 (32)	167 (31)	385 (31)	0.90
3	292 (13)	108 (20)	132 (11)	< 0.001
4	31 (1)	11 (2)	14 (1)	0.14
Missing	199 (8)	54 (10)	131 (11)	0.70
Cardiovascular diagnoses, n (%)				
0	755 (34)	158 (29)	428 (35)	0.03
1	823 (37)	203 (38)	433 (35)	0.32
2	449 (20)	113 (21)	242 (20)	0.53
≥3	222 (10)	65 (12)	128 (10)	0.30
Depression, n (%)	207 (9)	52 (10)	114 (9)	0.80
Anxiety, n (%)	196 (9)	44 (8)	123 (10)	0.23
Diabetes mellitus, n (%)	291 (13)	73 (14)	154 (13)	0.55
Renal failure, n (%)	97 (4)	28 (5)	57 (5)	0.60
Oral glucocorticoids, n (%)	1375 (61)	327 (61)	731 (60)	0.61

Data presented as mean ± SD unless otherwise specified. Hospitalizations and diagnoses were assessed within the four-year period before the start of long-term oxygen therapy (LTOT). *Abbreviations*: FEV_1_, forced expiratory volume in one second; PaO_2_, arterial blood gas tension of oxygen; PaCO_2_, arterial blood gas tension of carbon dioxide on air; WHO, world health organization.

### LTOT 24 vs 15 h/day

In the primary analysis among all patients (N = 2,249), higher daily LTOT duration analyzed as a continuous variable was not associated with any change in mortality rate, HR 1.00 (95% CI, 0.98 to 1.02) per 1 h/day increase above 15 h/day (Table 2). WHO performance status was found to be an important confounder (Table 2). Findings were similar for adjusted respiratory deaths (SHR 0.99; 95% CI, 0.97 to 1.02) and cardiovascular deaths (SHR 1.00; 95% CI, 0.96 to 1.05).

**Table 2:** Cox regression of daily oxygen duration and adjusted mortality in 2,249 patients with COPD.

Variable	Hazard ratio	95% CI	P-value
Continuous LTOT (24 vs. 15 h/day)	1.00	0.98–1.02	0.88
Age (per year)	1.04	1.03–1.05	< 0.001
Men	1.29	1.08–1.46	< 0.001
PaO_2_ air (per 1 kPa)	0.93	0.86–1.00	0.04
PaCO_2_ air (per 1 kPa)	-	-	0.001
PaCO_2_ air^†^	-	-	< 0.001
FEV_1_ (per liter)	0.96	0.80–1.15	0.65
Prescribed oxygen dose (per 1 l/min)	1.03	0.98–1.08	0.23
Body mass index, kg/m^2^			
< 18.5	1.37	1.16–1.63	< 0.001
18.5–24.9	Ref	-	-
25–29.9	0.73	0.60–0.87	0.001
≥ 30	0.80	0.64–1.00	0.06
WHO performance status			
0	*Ref*	-	-
1	1.03	0.75–1.40	0.88
2	1.51	1.10–2.07	0.01
3	2.45	1.76–3.42	< 0.001
4	3.19	1.93–5.28	< 0.001
Missing	1.35	0.94–1.93	0.10
Cardiovascular diagnoses			
0	*Ref*	-	-
1	1.26	1.09–1.46	0.002
2	1.40	1.18–1.66	< 0.001
≥3	1.35	1.08–1.67	0.007
Anxiety	1.28	1.05–1.58	0.01
Renal failure	1.33	1.03–1.73	0.03
Oral glucocorticoids	1.20	1.06–1.35	0.004

^†^PaCO_2_ air was included as second degree polynomial (Wald P < 0.001), wherefore a linear hazard ratio is not reported. *Abbreviations*: CI, confidence interval; for others see Table 1.

**Fig 1:**
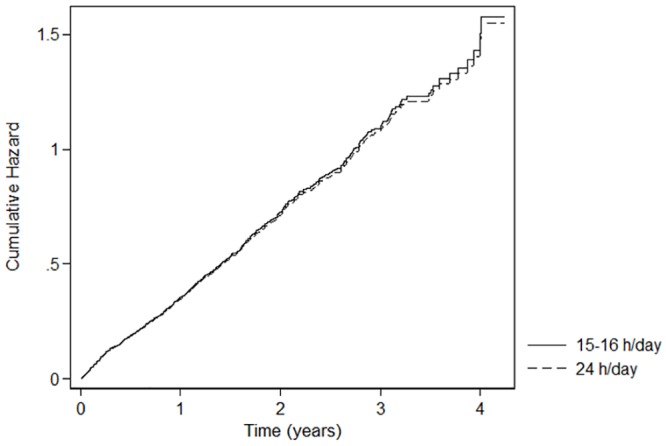
Cumulative risk of death for LTOT 24 h/day (N = 539) versus 15–16 h/day (N = 1,231) in oxygen-dependent COPD. The hazard ratio was 0.98 (95% CI, 0.85 to 1.14); adjusted for baseline age, sex, oxygen dose, PaO_2_ (air), PaCO_2_ (air), FEV_1_, WHO performance status, body mass index, treatment with oral glucocorticoids, and comorbid diagnoses including anxiety, renal failure and number of cardiovascular diagnoses.

In the secondary analysis (N = 1,770), LTOT 24 h/day was associated with a higher all-cause mortality rate than treatment 15–16 h/day, HR 1.17 (95% CI, 1.02 to 1.34) in unadjusted analysis. However, in the final model adjusted mortality was similar between LTOT 24 h/day and LTOT 15–16 h/day, HR 0.98 (95% CI, 0.85 to 1.14; P = 0.81). The adjusted hazard plots of the treatment groups closely overlapped at all time points (Fig 1).

In unadjusted analysis, there was a trend towards increased respiratory deaths (SHR 1.09; 95% CI, 0.89 to 1.34) and cardiovascular deaths (SHR 1.17; 95% CI, 0.81 to 1.71) with LTOT 24 h/day compared with LTOT 15–16 h/day. Adjusted cause-specific mortality was however similar between the two treatment groups for respiratory deaths (SHR 0.96; 95% CI, 0.76 to 1.20) and cardiovascular deaths (SHR 0.89; 95% CI, 0.60 to 1.32).

## Discussion

### Main finding

Longer daily duration of LTOT above 15 h/day was not associated with improved adjusted survival time in a large cohort of patients with oxygen-dependent COPD. Adjusted mortality rates were similar for deaths from all causes and from respiratory and cardiovascular disease.

### Strengths and limitations

Strengths of the present study include its national, population-based design and large study population. This study included 2,249 patients with COPD and therapeutic LTOT compared to a total of 280 patients in the randomized trials in severe hypoxemia.[1, 2] Analyses were adjusted for a range of relevant confounders, including blood gases, lung function, performance status, and comorbid diseases. Cause-specific mortality was analyzed as respiratory and cardiovascular disease are leading causes of death among these patients and may be affected by hypoxemia and supplemental oxygen therapy.[12]

A limitation is that we lacked data on actual daily oxygen utilization. Therefore, we cannot exclude the possibility that patients prescribed LTOT 24 h/day utilized oxygen fewer hours/day in real life and explain why similar rated of mortality was found in both groups. Our findings reflect effectiveness of prescribed oxygen durations in clinical practice. Adherence to LTOT may be insufficient but has been reported to be better in patients with significant hypoxemia like those in the present study.[16] A second limitation common to all observational designs is possible confounding by indication owing to the lack of randomization.[31] Patients with more severe illness may be prescribed higher oxygen flow rates and longer LTOT duration. In the present study, LTOT 24 h/day was associated with an increased mortality risk in the unadjusted analysis, but the survival difference disappeared when controlling for confounders including prescribed oxygen dose and WHO performance status. Our findings support our research hypothesis that a longer LTOT duration does not provide additional survival benefit above 15 h/day. However, absence of residual confounding needs to be evaluated through randomized controlled trials.

### What this study adds

This is the first large comparative study of LTOT prescribed 24 h/day versus treatment 15 h/day on survival in oxygen-dependent COPD. Our findings are made more representative and robust due to its population-based and multi-center design. The previous four studies of the effects of LTOT on mortality were small (in total 501 patients), conducted 20–40 years ago, totally un-blinded, and included selected patients at tertiary specialist centers.[1, 2] The included patients were younger, mostly men with no or limited comorbidity and did not receive modern treatments including for COPD and cardiovascular disease.[1–3, 32, 33] The dramatic survival benefit of LTOT seen in the 1970s was not evident in the studies from the 1990s.[1–3] The validity of the initial observations for today’s patients is unclear as the majority of current patients are women, elderly with multiple comorbidities and limited expected survival, similar to our study population.[5, 10–12] Our findings are in line with a recent small study (N = 228) which reported no survival difference between intermittent LTOT (mean utilization 6.7 ± 3.4 h/day) and continuous LTOT (mean utilization 18.1 ± 2.5 h/day).[34]

### Mechanisms

Whereas the survival benefit of LTOT 15 h/day or more is established,[1, 2] the mechanisms for the improvement in survival still remains unknown. There is limited evidence, but possible effects of LTOT might be mediated through prevention of hypoxemia-related cardiovascular disease and stabilization of pulmonary arterial pressure.[35] However, the effect on pulmonary arterial hypertension was found to be similar between for LTOT 15 h/day and 18 h/day.[36] LTOT prescribed 15 h/day might thus be sufficient which is in line with our findings of similar rates of cardiovascular mortality between LTOT prescribed 24 h/day compared with LTOT 15–16 h/day.

### Implications

The lack of association between LTOT 24 h/day and improved survival suggests that there is no advantage of LTOT prescribed 24 h/day compared with 15 h/day. On the contrary, LTOT 24 h/day may pose an unnecessary burden and limitation for many patients compared with treatment 15 h/day, where patients can be disconnected from the equipment for 9 hours each day. There is no consistent evidence that LTOT reduces breathlessness or health status in daily life.[37–40] LTOT has been associated with feelings of dependence and shame, which could contribute to a reluctance to leave the house, increased social isolation and deconditioning, especially with oxygen therapy given continuously 24 h/day.[13, 15, 17] Low-flow oxygen therapy has been associated with oxidative stress [41] and inflammation [42] which could contribute to increased morbidity and adverse health effects.[43, 44] LTOT 24 h/day is also associated with an increased electricity cost, which might be problematic for severely ill patients with already strained finances.

Current guidelines recommending continuous LTOT (optimally 24 h/day) are based on an observational, unadjusted comparison of the treatment arms from two randomized trials from the 1970s. [1, 2] [8] This recommendation is not evidence-based as no randomized study has compared the effect of LTOT 24 h/day with 15 h/day. The validity of LTOT among today’s patients is difficult to assess as comparisons with none or intermittent LTOT <15 h/day may be ethically unacceptable. In light of the present findings,[9] there is equipoise between LTOT prescribed 24 h/day and 15 h/day. We propose that a next step would be to validate the effect on survival of LTOT 24 h/day compared 15 h/day in a registry-randomized controlled trial (R-RCT).[45]

### Register-based randomized trial (R-RCT)

An R-RCT uses a health care registry to recruit, randomize, and follow-up patients in a clinical trial.[45] The R-RCT approach combines the features of a prospective randomized interventional trial with a large-scale clinical registry and thus enables evaluation of clinically important patient outcomes in the real world setting.[45] The strengths of an R-RCT include a simple design, fast enrolment, control of non-enrolled patients, being inexpensive and the possibility of very long-term follow up.[45] The method was recently pioneered in a randomized trial of thrombus aspiration during percutaneous coronary interventions, which included more than 60% of patients with acute ST-elevation myocardial infarction nationwide at low costs.[46] Hospitalization and mortality rate were assessed using national registries, with complete follow-up.[46]

An R-RCT using the National Swedevox register would be optimally suited for assessment of the effectiveness of continuous (24 h/day) versus low duration LTOT 15 h/day in a large representative sample of patients with chronic respiratory failure.

## Conclusions

LTOT prescribed 24 h/day was not associated with improved survival compared with LTOT 15–16 h/day in hypoxemic COPD after adjusting for potential confounders. The novel design of a registry-based randomized controlled trial is proposed to drive forward evidence-based care in patients with respiratory failure.

## Abbreviations

BMI: Body Mass Index
CI: Confidence Interval
COPD: Chronic Obstructive Pulmonary Disease
HR: Hazard Ratio
ICD: International Classification of Disease
IQR: Interquartile Range
FEV_1_: Forced Expiratory Volume in one second
FEV_1_% of predicted: Forced Expiratory Eolume in one second as percentage of predicted value
LTOT: Long-Term Oxygen Therapy
PaO_2_ air: Arterial blood gas tension of Oxygen on air
PaO_2_ oxygen: Arterial blood gas tension of Oxygen on oxygen
PaCO_2_ air: Arterial blood gas tension of Carbon dioxide on air
PaCO_2_ oxygen: Arterial blood gas tension of Carbon dioxide on oxygen
Ref: Reference category
SD: Standard Deviation
SHR: Subdistribution Hazard Ratios
WHO: World Health Organization
